# Herd immunity alters the conditions for performing dose schedule comparisons: an individual-based model of pneumococcal carriage

**DOI:** 10.1186/s12879-019-3833-6

**Published:** 2019-03-05

**Authors:** Alan Yang, Francisco Cai, Marc Lipsitch

**Affiliations:** 1 Harvard University, 677 Huntington Ave, Kresge Building, Room 506G, Boston, MA 02115 USA; 2 Harvard T.H. Chan School of Public Health, 677 Huntington Ave, Kresge Building, Room 506G, Boston, MA 02115 USA

**Keywords:** Pneumococcal, Conjugate, Vaccine, Trial, Dosage, Simulation, Herd, Immunity

## Abstract

**Background:**

There is great interest in the use of reduced dosing schedules for pneumococcal conjugate vaccines, a strategy premised on maintaining an acceptable level of protection against disease and carriage of the organism. We asked about the practicality of measuring differential effectiveness against carriage in a population with and without widespread use of the vaccine for infants.

**Methods:**

We adapted an existing transmission-dynamic, individual-based stochastic model fitted to the prevaccine epidemiology of pneumococcal carriage in the United States, and compared the observed vaccine-type carriage prevalence in different arms of a simulated trial with one, two, or three infant doses plus a 12-month booster. Using these simulations, we calculated vaccine efficacy that would be estimated at different times post-enrollment in the trial and calculated required sample sizes to see a difference in carriage prevalence.

**Results:**

In a pneumococcal conjugate vaccine (PCV)-naïve population, the difference in vaccine-type (VT) pneumococcal carriage prevalence between trial arms was less than 7% and varied with sampling time. In a population already receiving routine PCV administration, VT pneumococcal prevalence is nearly indistinguishable between trial arms. Relative efficacy of different dosing schedules was strongly dependent on the time between enrollment and sampling, with maximal prevalence differences reached 1–3 years post-enrollment. Moreover, vaccine efficacy estimates were typically slightly higher in trials in communities already receiving vaccination. Despite this, much larger sample sizes—by more than an order of magnitude—are required for a vaccine trial conducted in a population receiving routine PCV administration as compared to in a PCV-naïve population.

**Conclusions:**

These findings highlight some underappreciated aspects of clinical trials of pneumococcal conjugate vaccines with efficacy endpoints, such as the context- and time-dependence of efficacy estimates. They support the wisdom of conducting comparative dose schedule trials of conjugate vaccine effects on carriage in vaccine-naïve populations.

**Electronic supplementary material:**

The online version of this article (10.1186/s12879-019-3833-6) contains supplementary material, which is available to authorized users.

## Background

Mass vaccination of infants and toddlers with pneumococcal conjugate vaccines (PCVs) has led to large declines in pneumococcal disease in countries around the world [1]. These vaccines currently contain 10 or 13 different capsular polysaccharides from *Streptococcus pneumoniae*, each conjugated to a protein carrier. In *S. pneumoniae*, the chemical structure of the capsular polysaccharide determines the serotype, defined by the specificity of antibody responses against these capsules. PCVs induce immunity that directly protects recipients against vaccine serotypes (VT), which can cause invasive and mucosal disease [2]. It is clear that the immunity induced by PCVs also partially protects recipients against nasopharyngeal carriage of VT pneumococci [3]. This is important because the nasopharyngeal pneumococcal population is the source of transmission to other hosts, so the reduced carriage has led to herd immunity that has reduced VT disease in other age groups in many populations in which infants and/or toddlers have received PCVs [4, 5].

Dosing schedules adopted in national programs to date vary, with either 2 or 3 doses in infancy, with or without a booster around 12 months of age (schedules are referred to by the number of doses in infancy + the number of boosters, e.g. 2 + 1 or 3 + 0). The high market cost of PCVs has created intense interest in the possibility of reducing the total number of doses administered [6]. This interest was recently bolstered by a randomized, controlled trial showing comparable post booster immunogenicity in the UK for a 1 + 1 vs. a 2 + 1 schedule [7, 8]. However, few individually randomized trials (iRCTs) testing the impact of reduced dosing schedules on an efficacy outcome, such as prevalence of VT pneumococcal carriage, are carried out in countries that have already implemented routine vaccination. Indeed, besides a trial in South Africa [9], most of these iRCTs are being carried out in populations that have not yet introduced routine pneumococcal vaccination [10]. If these trials were to be conducted in countries with established vaccination programs, they would be challenged by the presence of herd immunity in the background population. We undertook simulation modeling of such iRCTs in newborns in both the presence and absence of background herd immunity to understand the effects of background herd immunity on the ability of a vaccine trial to reveal differences in efficacy against vaccine-type carriage between its arms. We found that the differences between arms in VT pneumococcal colonization in such a trial conducted in a background of herd immunity would be very small, and the resulting sample size requirement would be impracticably large. These findings support the wisdom of locating such trials with carriage outcomes in countries that have not yet introduced routine PCV programs.

## Methods

To simulate *S. pneumoniae* transmission dynamics, we used a discrete-time individual-based model based on a previous model developed by Cobey et al. [11]. As in Cobey et al.’s model, our model includes both serotype-specific and non-serotype-specific immunity and reproduces observed serotype diversity. The calculation of the force of colonization and duration of colonization also follow Cobey et al. In particular, the force of colonization for each individual is calculated every day with age-assortative mixing in transmission. This age-assortative mixing is parameterized by a polymod mixing matrix that was initially constructed from the Dutch population and is similar to that estimated for other western European countries [12]; we are unaware of a comparable dataset for the United States. Furthermore, individuals can be colonized by multiple strains of the same serotype at the same time, but the model is based on a “neutral null model” that avoids artificial predictions of serotype coexistence [13].

Our model differed from that of Cobey et al. in three ways. First, some versions of Cobey et al.’s model take into account a range of demographic events such as partnering, reproduction, and departure from the household of origin. By contrast, our model does not include these events because they are not central to the question we are interested in, which is the effect of herd immunity on the results of vaccine trials.

Second, our procedure for fitting model parameters deviated from that of Cobey et al.’s study. Cobey et al. estimated the total prevalence of pneumococcal carriage by averaging the annual samples in the final 10 years of their simulations, whereas we average over a longer period—the final 46 years—to obtain more precise estimates. The parameters were fit using 2001 data from the SPARC project, which sampled children aged 7 or younger in Massachusetts, USA and reported a 28% carriage prevalence [14]. We fit to serotype-specific prevalences using an algorithm previously described [15]. In brief, we iteratively update each serotype’s “fitness” parameter, which determines its competitive ability and duration of colonization, until its simulated prevalence (averaged over the final decades of each simulation run) matches its empirical prevalence in the SPARC data (Additional file 1: Figure S5). All 40 serotypes in the SPARC data were modeled. Sources of sampling error–sampling one serotype per individual and sampling a subset of the pediatric population–were not modeled. For simplicity, we assumed each serotype was equally likely to be sampled. Since the SPARC data is from 2001, only one year after the introduction of PCV7 in the United States, we elected not to model vaccine use when fitting the parameters.

The third difference was that we implemented a vaccine trial. In real-world individually-randomized vaccine trials, the effect of the trial on background transmission is minimized by keeping the size of the trial small relative to the general population: the assumption is that participants constitute a negligible fraction of the overall transmitting population and thus minimally affect transmission. In our simulations, we employed a computational strategy to circumvent this size requirement while still protecting the background population’s epidemiology from the members of the trial. To do this, we modeled participants in our vaccine trials by adding them as “ghost” individuals to the simulation who could receive colonizations from individuals in the general population but could not themselves transmit colonizations to any other individuals. This design allows us to introduce a large number of vaccine trial participants to the simulation—and thus obtain stable and precise estimates of the prevalence of VT colonization in the different arms—without interfering with the level of colonization in the background population. These prevalence estimates were then used as the “true” population means in our sample size calculations.

We simulated a vaccine trial with four arms receiving the 13-valent pneumococcal vaccine (PCV13) on different schedules—3 + 1, 2 + 1, 1 + 1, and unvaccinated (control)—where the booster was always given at 12 months of age and the primary sequence was at 2, 4, and 6 months of age, with the reduced schedules omitting the 6 month, or the 4 and 6 month doses respectively. These trials took place in one of two settings: first, in a setting where the 7-valent PCV (PCV7) had been in routine use for some years and had recently been replaced by PCV13 (see details below), and second, in a PCV-naïve population. Our simulation consisted of three sequential phases:

1. **Demographic Phase (50 years)**: There is no transmission and the goal is the reach an equilibrium age distribution in the background population mimicking that in the US population. Vaccine trial has not been initiated yet. Individuals’ ages and colonizations are recorded every year.
2. **Epidemiologic Phase (50 years)**: At the beginning of this phase, we introduce the disease to the population by randomly seeding each individual in the background population with colonizations and allowing transmission to take place and equilibrate. Initially, as a temporary measure, individuals are artificially given some pre-existing immunity to prevent a computationally intensive epidemic that would have otherwise occurred had the population been fully susceptible. If simulating in the presence of routine PCV vaccination, we begin introducing PCV7 to newborns in the background population 33 years into this phase. Then, 43 years into the phase, we replace PCV7 with PCV13 in the background population. This timeline was chosen to match pneumococcal vaccination in the USA, where PCV7 was administered for 10 years before switching to PCV13 [16]. In this phase of the simulation, the vaccine trial has not started yet. Individuals’ ages and colonizations are recorded every year.
3. **Vaccine Trial Phase (5 years)**: We begin the vaccine trial by introducing newborn participants to the simulation who are able to receive colonizations but unable to transmit them. If we are simulating the vaccine trial in the presence of herd immunity from PCV, the background population continues to receive PCV13. Individuals’ ages and colonizations are recorded more frequently—every month.

We simulated 50,000 individuals in the background population (our population size stayed constant, as in Cobey et al.) and an additional 10,000 “ghost” individuals in each of the 4 arms of the trial. Vaccines were assumed to confer varying degrees of protection with increasing numbers of doses, with the increased efficacy on subsequent doses taking effect immediately on receipt of the next dose. Vaccine efficacy after two or more doses (for 2 + 1 and 3 + 1 PCV13 schedules) was estimated using VT prevalence from Dagan et al. [17] and calculated as one minus the prevalence odds ratio as described in Rinta-Kokko et al. (Table 1) [18]. PCV7 efficacy was assumed to follow the same schedule as PCV13, but limited to the relevant serotypes. Vaccine efficacy after one dose (for all three PCV13 schedules) was estimated using a similar approach with data from Ota et al. [19]. To our knowledge, no data was available for the efficacy of the 1 + 1 schedule after the booster dose. Because initial analyses (data not shown) had found very large sample sizes for comparative trials, we made the assumption that would yield the greatest difference between arms and thus the smallest sample size: that efficacy remained unchanged after the booster dose in the 1 + 1 schedule. This assumption runs counter to our hypothesis that population immunity significantly mitigates detectable differences between vaccine trial arms with different dose schedules. By making this conservative assumption, therefore, we only bolster our ultimate findings supporting our hypothesis. Other equally plausible assumptions would have resulted in greater protection of this trial group and thus would have required even larger sample sizes to see a difference with other arms.

**Table 1:** Vaccine Schedule and Efficacies Modeled After Dagan et al. [17]

Age	PCV13 1 + 1	PCV13 2 + 1	PCV13 3 + 1	PCV7
at 2 mo	0.172	0.172	0.172	0.172
at 4 mo	0.172	0.270	0.270	0.270
at 6 mo	0.172	0.270	0.462	0.462
at 12 mo	0.172	0.501	0.501	0.501

In particular, we are aware of immunogenicity studies performed by Goldblatt et al. [8, 20] which give a somewhat different picture of differences between dose schedules than Dagan et al.’s efficacy studies Specifically, Goldblatt et al. find that the pre-boost vaccine efficacies of the 2 + 1 and 3 + 1 schedules are virtually the same and that the post-boost vaccine efficacies of all three dose schedules are also virtually the same. While the main results of this paper use parameters in line with Dagan et al.’s estimates, we also ran the simulation using modified vaccine efficacy parameters more aligned with Goldblatt et al.’s findings to contextualize and justify this choice (Table 2).

**Table 2:** Vaccine Schedule and Efficacies Modeled After Goldblatt et al. [8, 20]

Age	PCV13 1 + 1	PCV13 2 + 1	PCV13 3 + 1	PCV7
at 2 mo	0.172	0.172	0.172	0.172
at 4 mo	0.172	0.366	0.366	0.366
at 6 mo	0.172	0.366	0.366	0.366
at 12 mo	0.501	0.501	0.501	0.501

**Table 3:** Summary of Sources for Model Parameters

Parameter	Source (country of origin)
Age-assortative polymod mixing matrix	Mossong et al. [12] (Netherlands)
Pre-vaccine serotype-specific carriage	Croucher et al. [14] (USA)
PCV introduction schedule	McLaughlin et al. [16] (USA)
Vaccine efficacy	• Main study: efficacy data from Dagan et al. [17] (Israel) • Supplemental figures: immunogenicity data from Goldblatt et al. [8, 20] (United Kingdom)

Since it does not appear that different vaccine parameters lead to unexpectedly different results, we could reasonably choose to follow either Dagan et al. or Goldblatt et al. in our main study. We decided to use the Dagan et al. parameters for three reasons. The first is that using a parameterization in which the 2 + 1 and 3 + 1 schedules are essentially identical (as in Table 2) would not be useful for examining the question of whether carriage differences between trial arms are harder to detect in immune populations. The second reason is that by assuming larger differences between different dose schedules, we are making an assumption that works against our hypothesis and thereby strengthening our ultimate findings if they support our hypothesis. Finally, another consideration was the fact that Dagan et al.’s results represent actual efficacy data whereas Goldblatt et al.’s findings do not. These three considerations motivated our decision to follow primarily Dagan et al.’s parameters, while keeping in mind that there continues to be debate on this topic: the difference in efficacy between the 2 + 1 and 3 + 1 schedules was not statistically significant in the Dagan et al. study, nor did the study include a 1 + 1 trial arm to confirm the assumption that the booster response is absent in this schedule.

With the parameters set (Table 3), we ran each simulation 50 times and averaged the results of these iterations to obtain fairly smooth prevalence estimates. The simulation was coded in C++ 11 and run using the software XCode (Version 7.2.1). Data analysis was done in Jupyter Notebook (Version 4.0.6).

We calculated the sample sizes needed to achieve a certain power in distinguishing two trial arms by taking the effect size to be the maximum difference in VT prevalence between the two trial arms during the course of the entire trial. We then determined the relationship between power and sample size by interpreting the VT prevalence of each trial as a proportion and by testing for superiority. We used Cohen’s effect size *h* to calculate the sample size *n* according to the following formula below [21]. The alternative hypothesis was that the trial arm with more doses will show a smaller VT prevalence than the other arm; this was tested against the null hypothesis that the two trial arms yield equivalent VT prevalences.

$$ n\kern0.5em =\kern0.5em 2{\left(\frac{z\beta + z\alpha}{h}\right)}^2\kern0.5em \mathrm{where}\kern0.5em h=2{\sin}^{-1}\left(\sqrt{p_1}\right)-2{\sin}^{-1}\left(\sqrt{p_2}\right). $$

*z*_*β*_ and *z*_*α*_ are defined as usual, namely the *z*-scores for Type II and Type I errors, respectively. *p*_1_ and *p*_2_ are the sample proportions.

In addition to sample size, we calculated what estimate of relative efficacy would emerge at different time points in the trial. Relative efficacy was defined as either one minus the risk ratio, or one minus the odds ratio, of VT carriage prevalence between arms of the trial, with the 1 + 1 arm as the reference group and the higher-dose groups as the “interventions.”

## Results

**Fig. 1:**
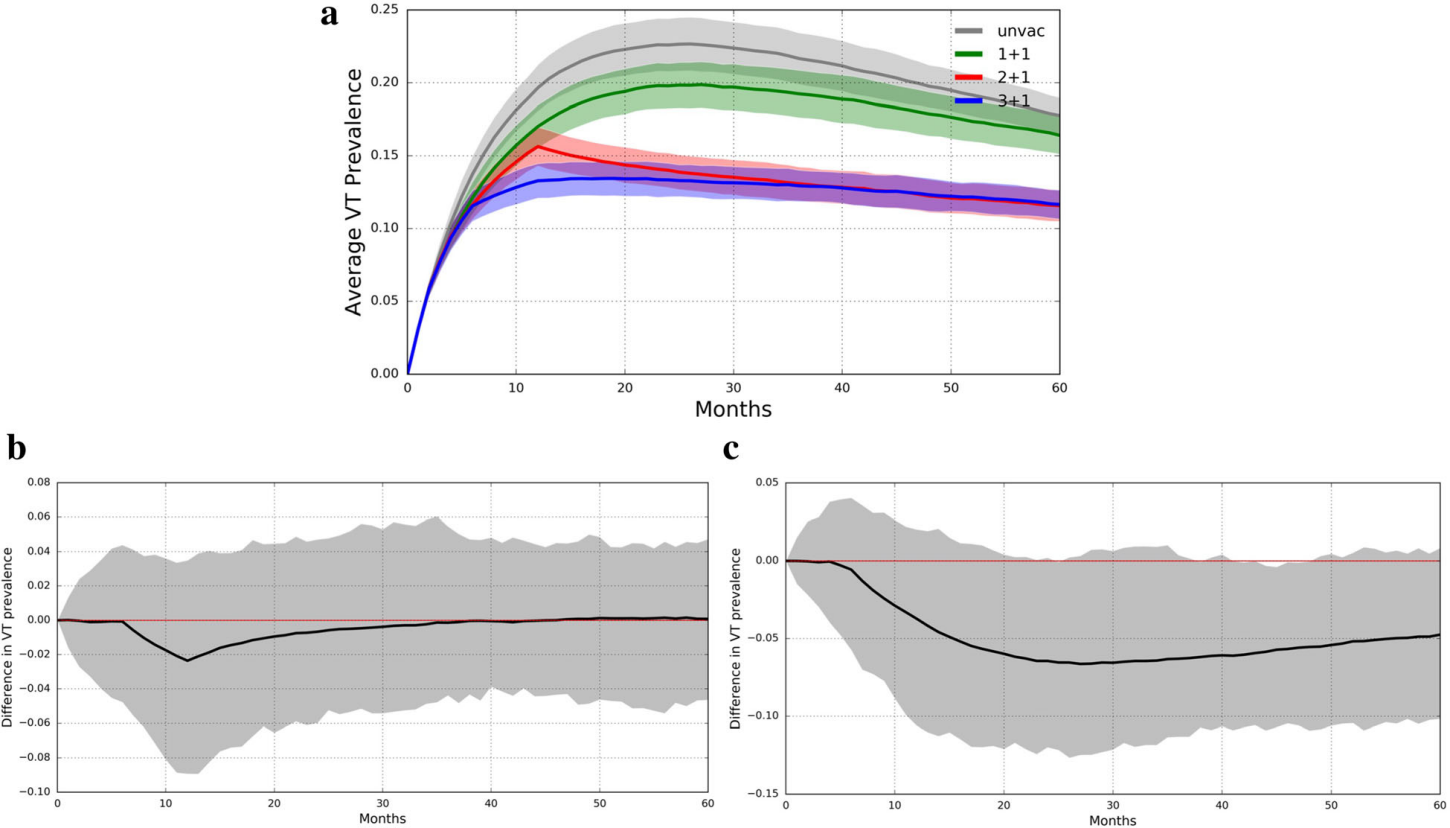
PCV13 Trial Simulated in Naïve Population. **a** VT carriage prevalence in all four trial arms (mean ± s.d.). Lines represent average VT prevalence across 50 simulations while shading is bounded by one standard deviation. **b** The difference in VT prevalence between the 3 + 1 and 2 + 1 trial arms (averaged across 50 simulations) graphed over time. **c** The difference in VT prevalence between the 3 + 1 and 1 + 1 trial arms (averaged across 10 simulations) graphed over time. The zero difference line is shown in red

In a PCV-naïve population, the difference in VT pneumococcal carriage prevalence between trial arms was less than 7% and varied with sampling time (Fig. 1a-c). The largest prevalence difference between the 3 + 1 and 1 + 1 trial arms, 6.6%, was observed 27 months after the start of the vaccine trial. Meanwhile, the largest difference between the 3 + 1 and the 2 + 1 trial arms was 2.4%, occurring 12 months into the vaccine trial. Under assumptions of less divergence in the efficacies of different dose schedules, however, the prevalences among the trial arms are more similar (Additional file 2: Figure S1). In fact, there is virtually no difference between the 2 + 1 and 3 + 1 schedules, as expected from their identical efficacy parameters. Moreover, the largest difference observed when comparing the 3 + 1 schedule with the 1 + 1 schedule occurs at 12 months due to identical post-boost assumed efficacies.

**Fig. 2:**
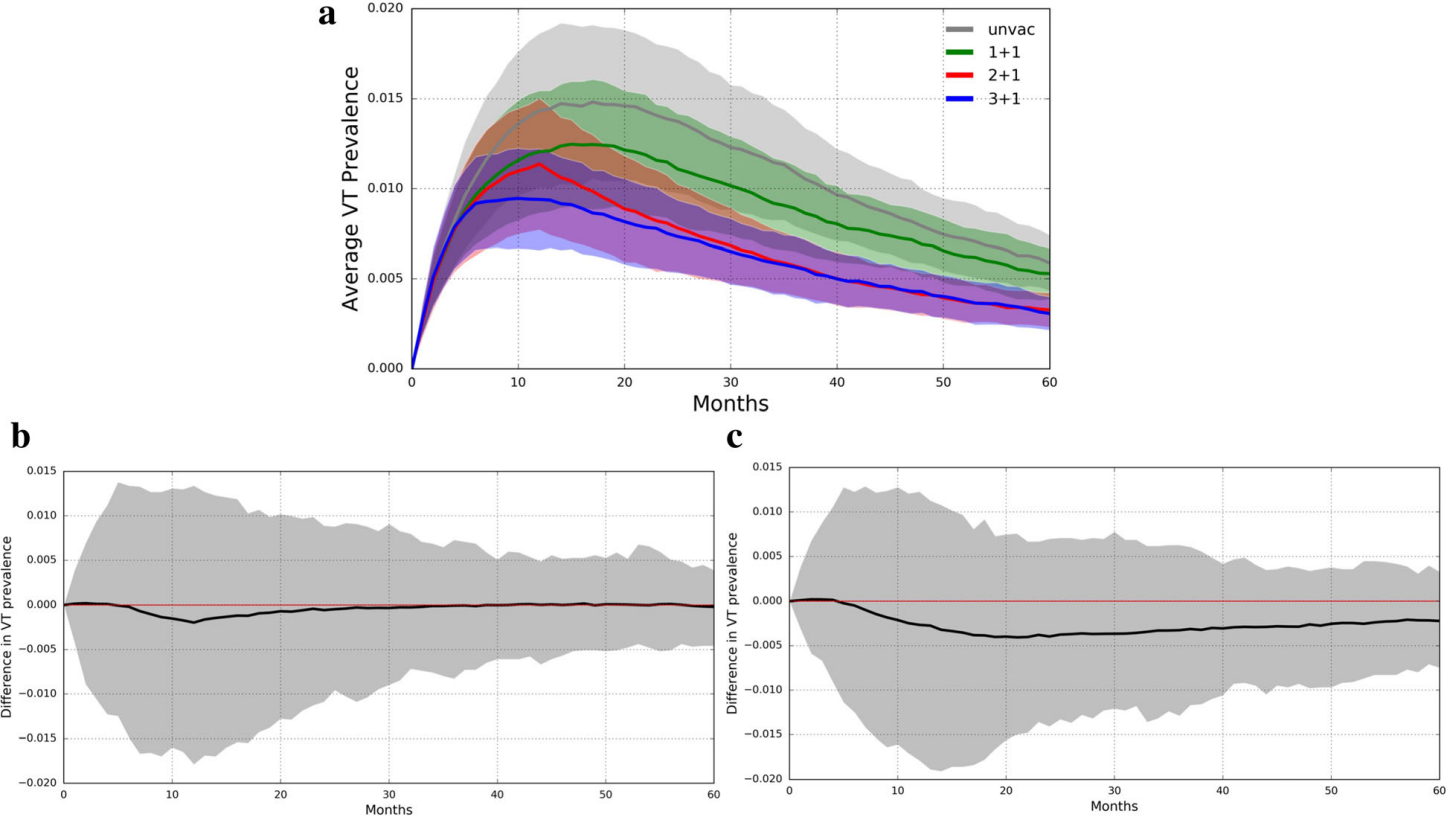
PCV13 Trial Simulated in 3 + 1 Vaccinated Population. **a** VT carriage prevalence in all four trial arms. Lines represent average VT prevalence across 50 simulations while shading is bounded by one standard deviation. **b** The difference in VT prevalence between the 3 + 1 and 2 + 1 trial arms (averaged across 50 simulations) graphed over time. **c** The difference in VT prevalence between the 3 + 1 and 1 + 1 trial arms (averaged across 50 simulations) graphed over time. The zero difference line is shown in red. Note the scale of the y-axis

In a population already receiving routine PCV administration, VT pneumococcal prevalence is nearly indistinguishable between trial arms (Fig. 2a-c). The overall prevalences in each trial arm are much lower than in a PCV-naïve population, and the differences between the trial arms are very small. Indeed, the largest difference between the 3 + 1 and 1 + 1 trial arms was 0.4%, which occurred 21 months into the vaccine trial. The largest difference between the 3 + 1 and 2 + 1 trial arms was only 0.2% and occurred 12 months into the vaccine trial. Here again, under assumptions of less divergence in the efficacies of different dose schedules, the prevalences among the trial arms are more similar (Additional file 3: Figure S2). There is again virtually no difference between the 2 + 1 and 3 + 1 schedules, and the maximum difference between the 3 + 1 and 1 + 1 arms occurs at 12 months.

**Fig. 3:**
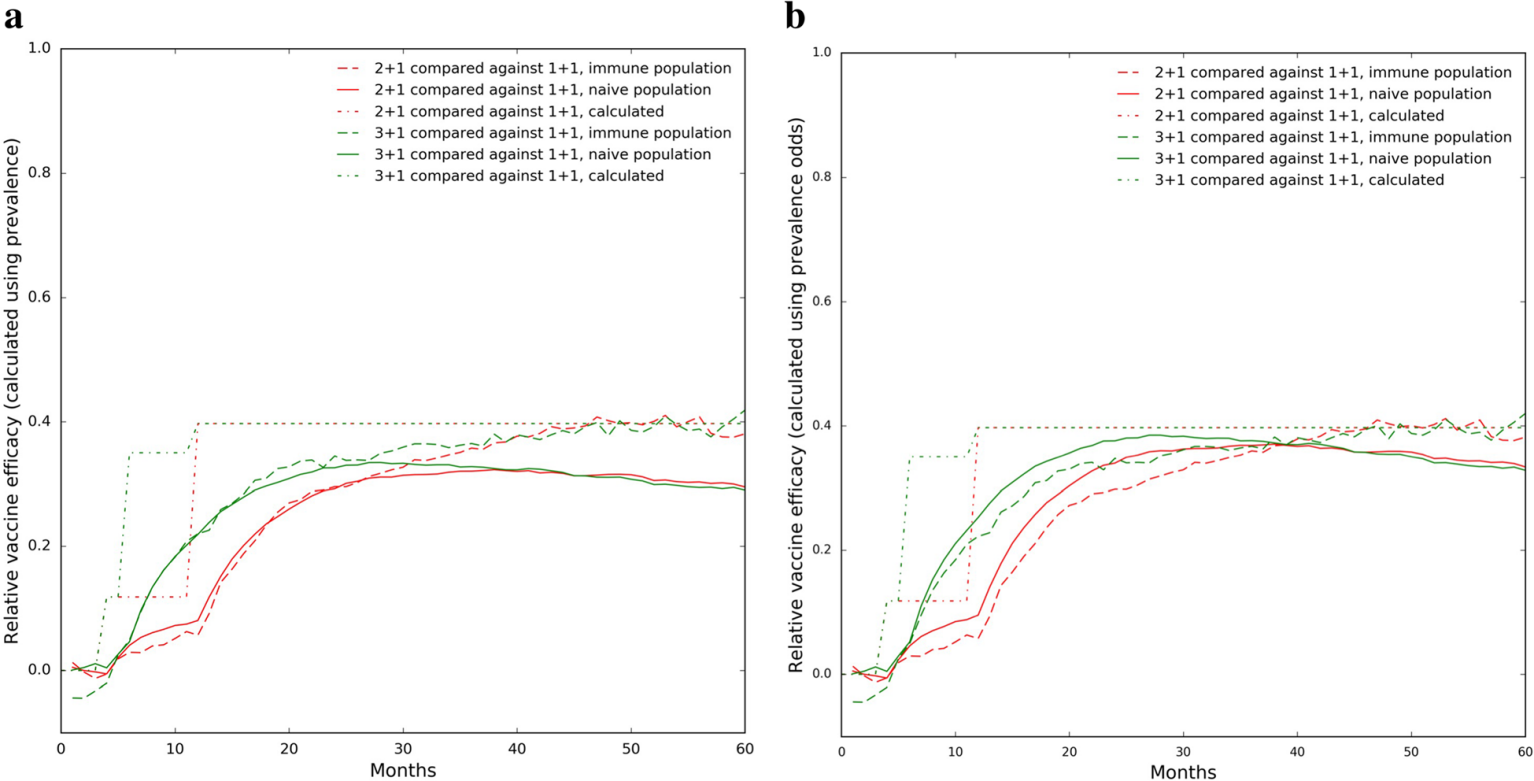
Relative Vaccine Efficacies in a Simulated PCV13 Trial. **a** Relative VE determined using prevalences. **b** Relative VE determined using prevalence odds. In both sub-figures, the “calculated” relative VE was determined from the initial VE parameters of the simulation according to the formula: Relative VE = 1 – (1 – VE_intervention_)/(1 – VE_reference_)

Relative vaccine efficacy estimation (Fig. 3). Relative vaccine efficacy (VE) against carriage is often calculated as VE = 1 – (prevalence of VT carriage in vaccinees)/(prevalence of VT carriage in controls), or alternatively as 1 – (prevalence odds of VT carriage in vaccinees)/(prevalence odds of VT carriage in controls) [1]. Figure 3 compares relative VE by each of these two measures over time, during the vaccine trial. Notably, relative VE varies with time since vaccination, due to the dynamic nature of pneumococcal colonization [15]. Furthermore, the relative efficacies of the 2 + 1 schedule and the 3 + 1 schedule diverged after 6 months but later converged again, as expected from the results in Fig. 1. Also interestingly, the relative VE measured post-vaccination was generally lower in a vaccine-naïve community than in a community already using PCV13. Moreover, this difference in VE grows larger as time goes on. However, it also should be noted that the relative VE, when calculated using prevalence odds, is actually slightly higher in a vaccine-naïve community than in an immune community during the first ~ 40 weeks of the trial (Fig. 3b).

For reference, we added curves to Fig. 3 representing relative VEs that are not derived through prevalence measurements but calculated from the simulation’s initial VE parameters. We calculated these curves by taking relative VE(t) = 1 – (1 – VE_intervention_(t)) / (1 – VE_reference_(t)) where *t* is time. These calculated curves therefore assume that the protection received from a dose is acquired instantaneously and stays constant thereafter. Besides the instantaneous nature of the changes in relative VE, these calculated curves closely matched our observed results. Moreover, the calculated relative VE matched the observed relative VEs in immune populations slightly better than in vaccine-naïve communities.

When the simulation is run with vaccine efficacy parameters following Goldblatt et al., the general trend that relative VEs measured in immune populations are higher still holds. However, one difference is that the relative VEs of the 3 + 1 and 2 + 1 dose schedules closely match each other throughout the entire trial rather than just towards the end, which makes sense since the two schedules are parameterized virtually identically. Another striking difference is that the relative VEs all converge to zero over time, which again is a result of the identical post-boost efficacies of all three dose schedules (Additional file 4: Figure S3).

**Fig. 4:**
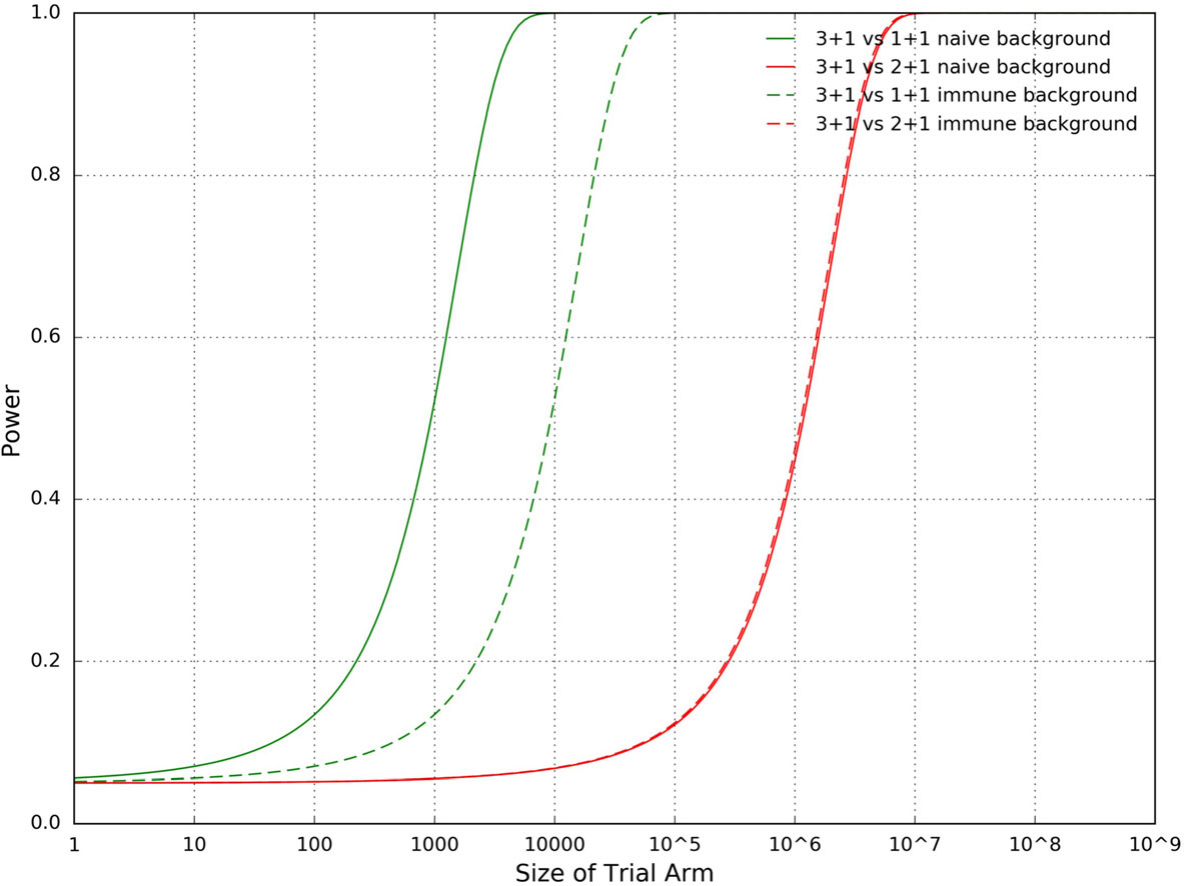
Power vs. Trial Arm Size in a Simulated PCV13 Trial

Much larger sample sizes—by more than an order of magnitude—are required for a vaccine trial conducted in a population receiving routine PCV administration as compared to in PCV-naïve population (Fig. 4). In a PCV-naïve population the sample size needed to distinguish the 3 + 1 arm from the 1 + 1 arm with a power of 80% is roughly 487 (*α* = 0.05); distinguishing the 3 + 1 arm from the 2 + 1 arm with a power of 80% requires a sample size of 3479 (again, *α* = 0.05). By contrast, in a population already receiving routine PCV administration, the sample size needed to distinguish the 3 + 1 arm from the 1 + 1 arm with a power of 80% is about 9358; distinguishing the 3 + 1 arm from the 2 + 1 arm here with a power of 80% requires a sample size of nearly 41,438. When the simulation is run with less divergence in efficacy between the different dose schedules, overall sample sizes required are higher, as expected (Additional file 5: Figure S4).

## Discussion

This simulation study showed that in the presence of significant herd immunity conferred by widespread uptake of PCVs, a clinical trial to estimate the effects of reduced dosing schedules on VT pneumococcal carriage would require impracticably large sample sizes. This occurs both because the level of VT carriage is reduced in the target age group and because of herd immunity effects.

These findings confirm the wisdom of the decision to test immunogenicity of reduced dosing schedules, but not efficacy against carriage, in countries that have already adopted PCV vaccination as a national policy [8]. By contrast, head-to-head randomized comparisons of dosing schedules should be conducted only in countries which have not yet introduced a policy of PCV use, such as Viet Nam [3, 22].

The result that it would be difficult to detect a small reduction in immune protection in a context of rare VT colonization was perhaps predictable by simple intuitive reasoning. However, previous work [15] has shown that pneumococcal vaccine trials with a colonization prevalence endpoint have several particularities that differ from those of more traditional vaccine trials using incidence of an acute infection as an endpoint [15, 23–26]. In particular, these studies have shown that several factors complicate the interpretation of such studies:

- the competition between different serotypes to colonize [26, 27]
- the ability of most assays to detect only one serotype among those colonized with one or more serotypes [28]
- the nonlinear relationship between incidence rate reduction (the biological parameter assumed to be relatively consistent for the same vaccine in different settings) and prevalence reduction (which is measured in such trials) [18]
- the fact that as children are receiving vaccine doses they are also acquiring immunity in both serotype-specific and nonspecific fashions to colonization [15].

Beyond this basic result, several aspects of our findings are noteworthy in the broader context of trial design and interpretation for trials with a carriage endpoint. For example, our finding that relative vaccine efficacy as measured by reduction in either risk or odds of carriage is higher in the presence of herd immunity runs counter to the usual pattern in vaccine trials using incidence as an endpoint, wherein reduced incidence in all trial arms may occur due to herd immunity, but the relative reduction in incidence due to vaccination (or more vaccination) is assumed to be constant across settings [29]. This finding may be understood as the consequence of at least two features of carriage-endpoint trials. First, the endpoint of carriage positivity (prevalence) in a setting, like this one, where multiple strains may be carried simultaneously, is not exactly proportional to incidence, the natural history parameter affected by the vaccine. An individual carrying two vaccine-type strains will be indistinguishable from an individual carrying only one vaccine-type strain by the carriage prevalence measure (assuming neither is carrying a nonvaccine type strain), yet the effect of the vaccine in a high-incidence setting will be partly to make some who would have had the former state, instead have the latter state. This effect will be smaller as vaccine-type incidence declines, thus will attenuate the observed vaccine effect to a lesser degree. Second, as control participants are exposed to vaccine-type carriage, they will develop some serotype-specific natural immunity, and this will happen more in a naïve than in an already-vaccinated population. Thus the relative protection from the vaccine will be less in a naïve population. This phenomenon also partly explains the strong time-dependence of measured relative efficacy of various dosing schedules, as efficacy first increases as existing colonization (not affected by the vaccine) is cleared and new colonization events are prevented, then declines as controls become partially immune in a serotype-specific fashion due to natural colonization, shrinking the difference between vaccine recipients and controls [15]. In general, simulations of clinical trials for vaccines or other infectious disease prevention measures can enhance intuition and improve study design [30].

Like any model, the simulation model here has several limitations. While it attempts to incorporate the major determinants of both serotype-specific (antibody-based, acquisition-reducing) and serotype-transcending (Th17-based, duration-reducing) immunity to carriage of pneumococci, as well as competition between different strains to colonize the same host, the design of the model required a number of simplifying assumptions, in particular that these were the only relevant forms of constraint on acquisition of pneumococcal carriage. We did not incorporate household structure in these simulations, because earlier work [11] showed that qualitative patterns were not strongly affected by these details. Carriage prevalence by serotype was calibrated to data from Massachusetts, USA [31], and the details of carriage prevalence overall can vary considerably across populations, though the leading serotypes (prior to vaccine introduction) are remarkably consistent. The assumed reductions in carriage incidence due to various dosing schedules were point estimates taken from a single study, and these are subject both to statistical uncertainty and possible variation across settings [11]. Despite these limitations, we believe that the large magnitude of the simulated difference between vaccine-experienced and vaccine-naïve settings shows robustly that such trials would be almost certainly underpowered if conducted in populations that have already introduced mass pneumococcal conjugate vaccination. Moreover, particularly in light of recent studies of a different vaccine and its likely performance in clinical trials, the finding of time-dependent variation in efficacy appears to be a robust result [15]. Finally, we calculated sample sizes for the point of largest prevalence difference in the simulations, for a superiority study. Sample sizes for a noninferiority study may differ depending on the margin of noninferiority chosen.

We further recognize that the results of our simulation, like those of any simulation, are dependent on the parameters used. In particular, in this study we found that modifying vaccine efficacy parameters to match the findings of Goldblatt et al.—namely, that pre-boost immunogenicity is virtually the same for 2 + 1 and 3 + 1 schedules and that post-boost immunogenicity is virtually the same for all three schedules—yields different results. Importantly, however, all of these differences could be fully explained by the differences in VE parameterization. In fact, the key result still held: when two trial arms are parameterized differently, the resulting difference in carriage is smaller when measured in an immune population, which means that larger sample sizes are needed to detect that difference. Our re-run of the simulation using different parameters demonstrates, therefore, that our results should be interpreted with the understanding that the parameters directly influence the data. Nonetheless, our simulation as currently parameterized has allowed us to make important analyses.

Our work has focused on dose schedule comparisons made from individually randomized, controlled trials in different contexts. To understand the long-term implications of switching a population from one vaccine schedule to another, further work is needed as, over time, persons who received reduced schedules as infants constitute a growing proportion of the population.

## Conclusions

This study demonstrates computationally that the presence of background herd immunity challenges the comparison of PCV dose schedules in a clinical trial. In particular, immune populations require impractical samples sizes that are more than an order of magnitude larger than those for vaccine-naïve populations in order to distinguish between the carriage prevalences of the arms of a PCV dosage-comparison trial. This result holds true, moreover, despite the fact that VE estimates are generally higher in immune populations than in vaccine-naïve populations.

Not only are the results of a PCV dose schedule comparison trial affected by background herd immunity, but they are also time-dependent. Specifically, the relative efficacy of different dosing schedules varies strongly with time, with maximal prevalence differences attained 1–3 years into the trial.

By highlighting the context- and time-dependence of efficacy estimates in PCV dose schedule comparison trials, these findings underscore some underappreciated aspects of these trials and support the wisdom of comparing differences in carriage between individuals receiving different dosages of PCV only in vaccine-naïve populations.

## Additional files

Additional file 1: **Figure S5.** Serotype-specific Prevalence Distribution After Fitting Model. Prevalence of each serotype is graphed over the number of iterations the model is fit to SPARC 2001 data. Each subplot is labeled with the serotype name and its fitness rank in our model. Target prevalences are shown in dotted lines. (JPG 657 kb)

Additional file 2: **Figure S1.** Simulated Trial of Goldblatt et al. PCV13 in a Naïve Population. Vaccine trial in naïve population simulated under assumptions of less divergence between the efficacies of different dose schedules. (a) VT carriage prevalence in all four trial arms (mean ± s.d.). Lines represent average VT prevalence across 50 simulations while shading is bounded by one standard deviation. (b) The difference in VT prevalence between the 3 + 1 and 2 + 1 trial arms (averaged across 50 simulations) graphed over time. (c) The difference in VT prevalence between the 3 + 1 and 1 + 1 trial arms (averaged across 10 simulations) graphed over time. The zero difference line is shown in red. (JPG 312 kb)

Additional file 3: **Figure S2.** Simulated Trial of Goldblatt et al. PCV13 in a 3 + 1 Vaccinated Population. Vaccine trial in 3 + 1 vaccinated population, simulated under assumptions of less divergence between the efficacies of different dose schedules. (a) VT carriage prevalence in all four trial arms. Lines represent average VT prevalence across 50 simulations while shading is bounded by one standard deviation. (b) The difference in VT prevalence between the 3 + 1 and 2 + 1 trial arms (averaged across 50 simulations) graphed over time. (c) The difference in VT prevalence between the 3 + 1 and 1 + 1 trial arms (averaged across 50 simulations) graphed over time. The zero difference line is shown in red. Note the scale of the y-axis. (JPG 309 kb)

Additional file 4: **Figure S3.** Relative Vaccine Efficacies of Goldblatt et al. PCV13 in a Simulated Trial. Relative vaccine efficacies during a vaccine trial simulated under assumptions of less divergence between the efficacies of different dose schedules. (a) Relative VE determined using prevalences. (b) Relative VE determined using prevalence odds. In both sub-figures, the “calculated” relative VE was determined from the initial VE parameters of the simulation according to the formula: Relative VE = 1 – (1 – VE_intervention_)/(1 – VE_reference_). (JPG 332 kb)

Additional file 5: **Figure S4.** Power vs. Trial Arm Size in a Simulated Trial of Goldblatt et al. PCV13. Simulated under assumptions of less divergence between the efficacies of different dose schedules. Sample sizes for a 3 + 1 vs 2 + 1 trial are not calculated because doing so would be incoherent; the arms are essentially identical, which implies an infinite theoretical sample size. (JPG 225 kb)

## Abbreviations

iRCT: individually randomized controlled trial
PCV: Pneumococcal conjugate vaccine
PCV13: 13-valent pneumococcal conjugate vaccine
PCV7: 7-valent pneumococcal conjugate vaccine
VE: vaccine efficacy
VT: Vaccine type
