# Supplementary figures and images for: Herd immunity alters the conditions for performing dose schedule comparisons: an individual-based model of pneumococcal carriage
Source: BMC Infect Dis. 2019 Mar 5;19:227. doi: 10.1186/s12879-019-3833-6 (PMC6402138; doi:10.1186/s12879-019-3833-6)

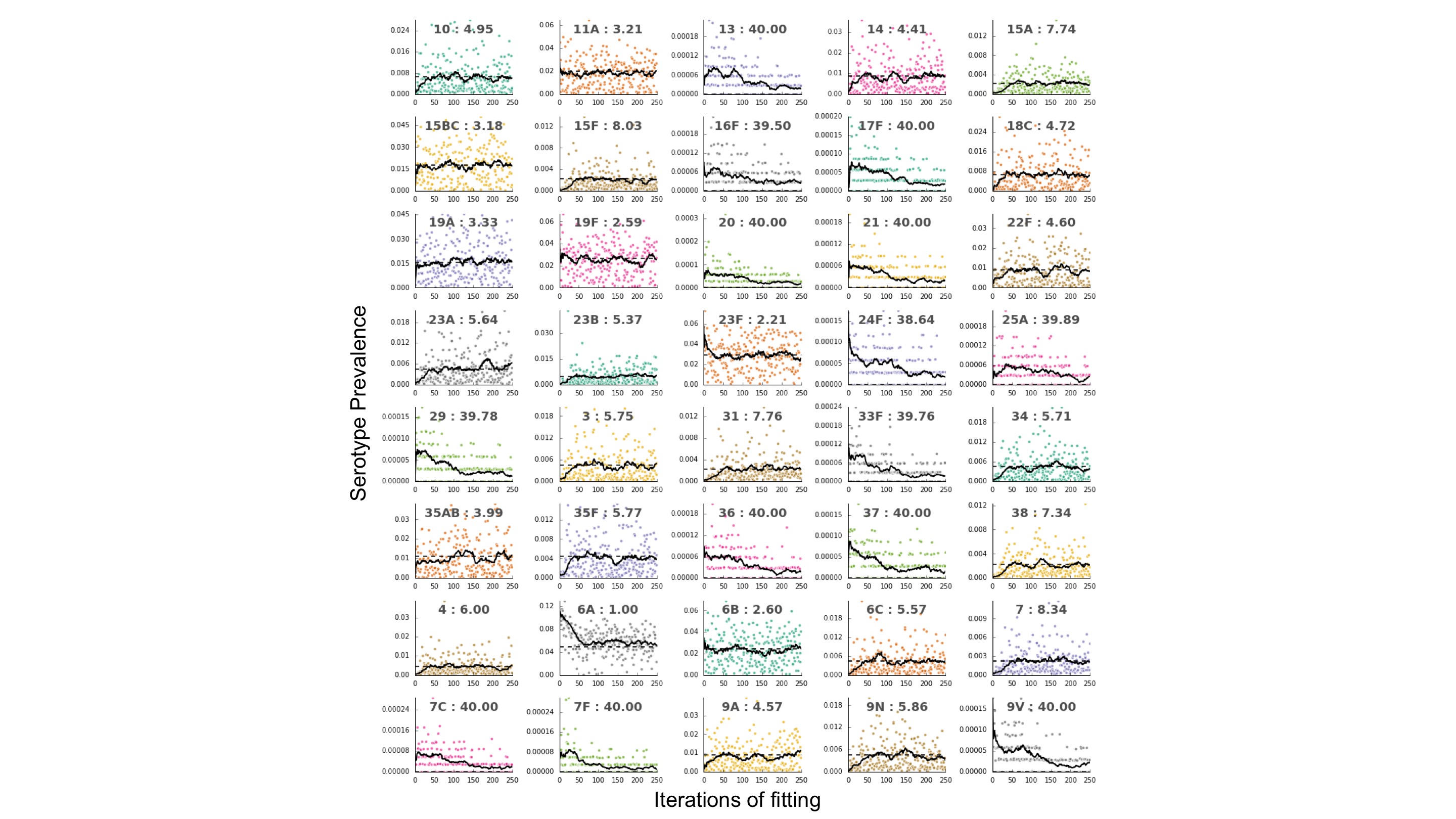

Supplement: Supplementary file 1 — Figure S5. Serotype-specific Prevalence Distribution After Fitting Model. Prevalence of each serotype is graphed over the number of iterations the model is fit to SPARC 2001 data. Each subplot is labeled with the serotype name and its fitness rank in our model. Target prevalences are shown in dotted lines. (JPG 657 kb) [file 12879_2019_3833_MOESM1_ESM.jpg]

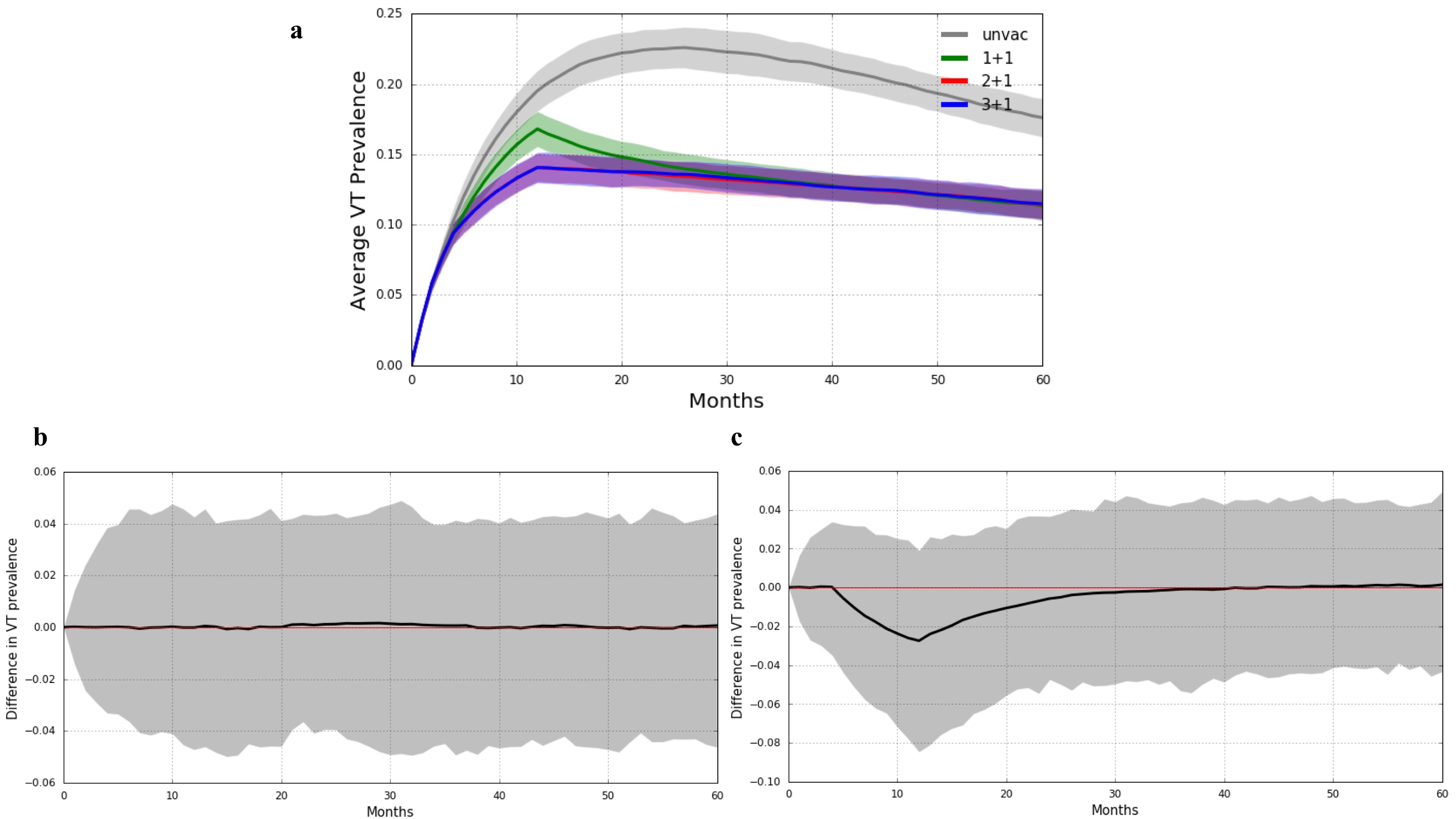

Supplement: Supplementary file 2 — Figure S1. Simulated Trial of Goldblatt et al. PCV13 in a Naïve Population. Vaccine trial in naïve population simulated under assumptions of less divergence between the efficacies of different dose schedules. (a) VT carriage prevalence in all four trial arms (mean ± s.d.). Lines represent average VT prevalence across 50 simulations while shading is bounded by one standard deviation. (b) The difference in VT prevalence between the 3 + 1 and 2 + 1 trial arms (averaged across 50 simulations) graphed over time. (c) The difference in VT prevalence between the 3 + 1 and 1 + 1 trial arms (averaged across 10 simulations) graphed over time. The zero difference line is shown in red. (JPG 312 kb) [file 12879_2019_3833_MOESM2_ESM.jpg]

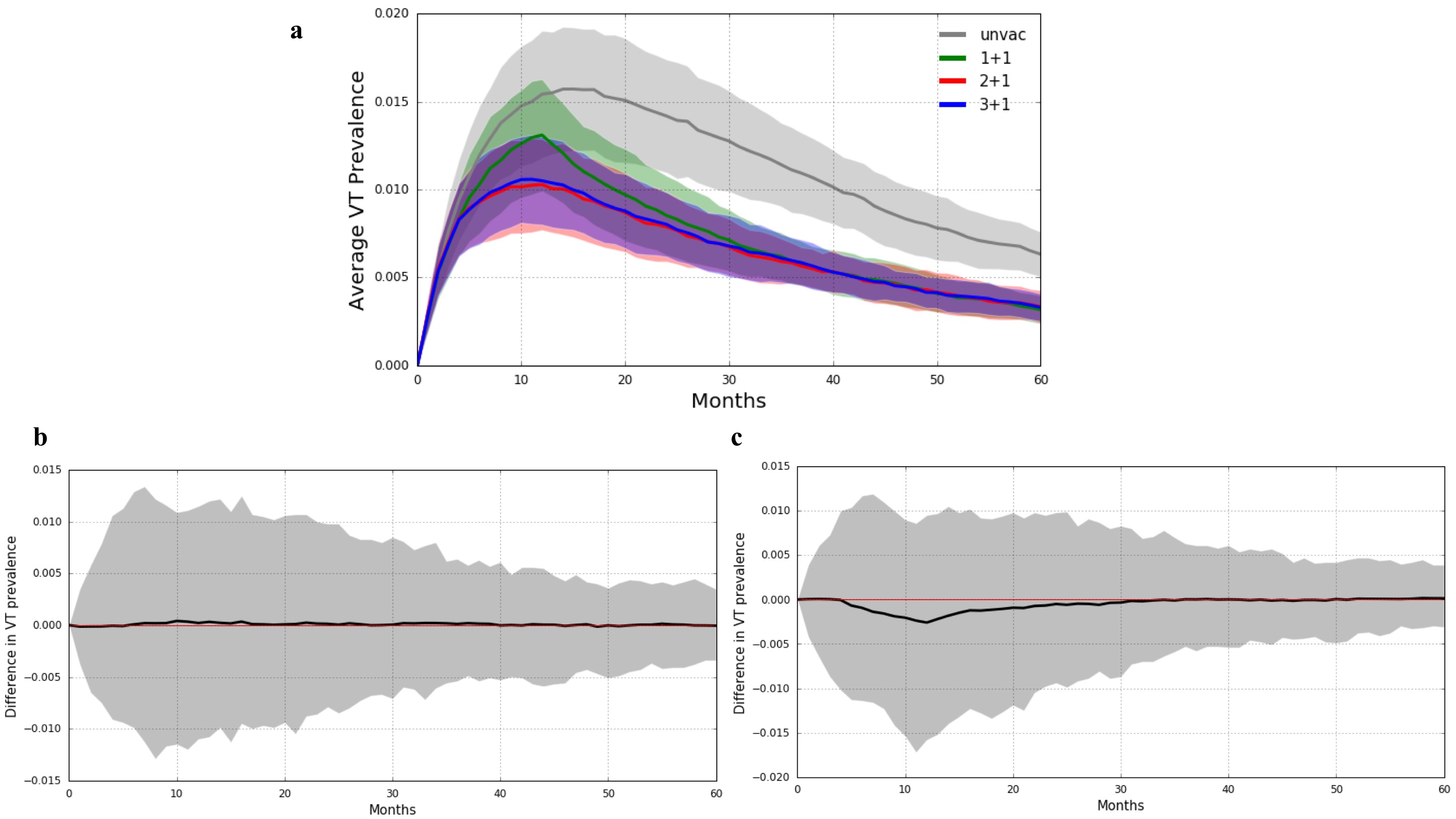

Supplement: Supplementary file 3 — Figure S2. Simulated Trial of Goldblatt et al. PCV13 in a 3 + 1 Vaccinated Population. Vaccine trial in 3 + 1 vaccinated population, simulated under assumptions of less divergence between the efficacies of different dose schedules. (a) VT carriage prevalence in all four trial arms. Lines represent average VT prevalence across 50 simulations while shading is bounded by one standard deviation. (b) The difference in VT prevalence between the 3 + 1 and 2 + 1 trial arms (averaged across 50 simulations) graphed over time. (c) The difference in VT prevalence between the 3 + 1 and 1 + 1 trial arms (averaged across 50 simulations) graphed over time. The zero difference line is shown in red. Note the scale of the y-axis. (JPG 309 kb) [file 12879_2019_3833_MOESM3_ESM.jpg]

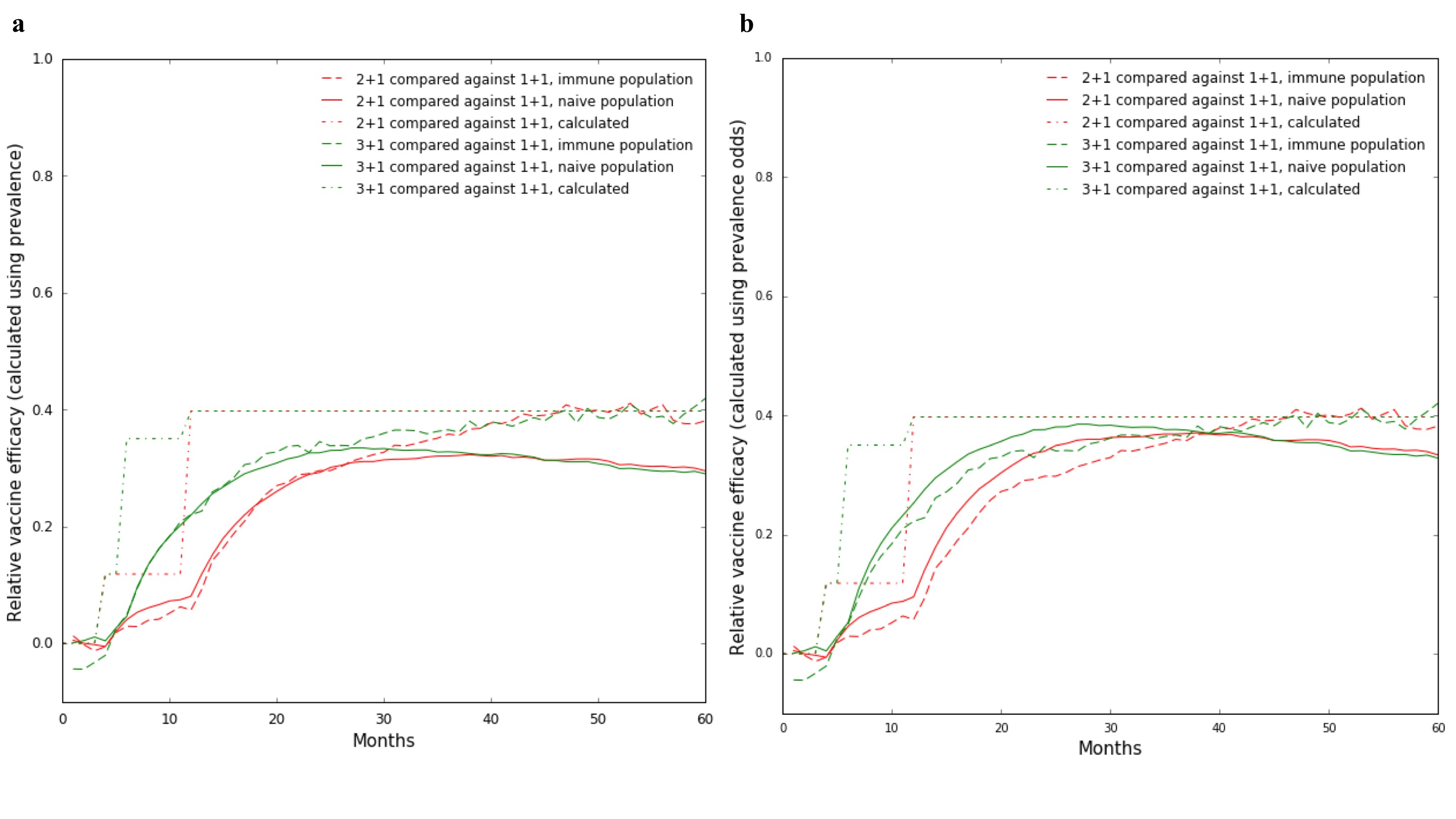

Supplement: Supplementary file 4 — Figure S3. Relative Vaccine Efficacies of Goldblatt et al. PCV13 in a Simulated Trial. Relative vaccine efficacies during a vaccine trial simulated under assumptions of less divergence between the efficacies of different dose schedules. (a) Relative VE determined using prevalences. (b) Relative VE determined using prevalence odds. In both sub-figures, the “calculated” relative VE was determined from the initial VE parameters of the simulation according to the formula: Relative VE = 1 – (1 – VEintervention)/(1 – VEreference). (JPG 332 kb) [file 12879_2019_3833_MOESM4_ESM.jpg]

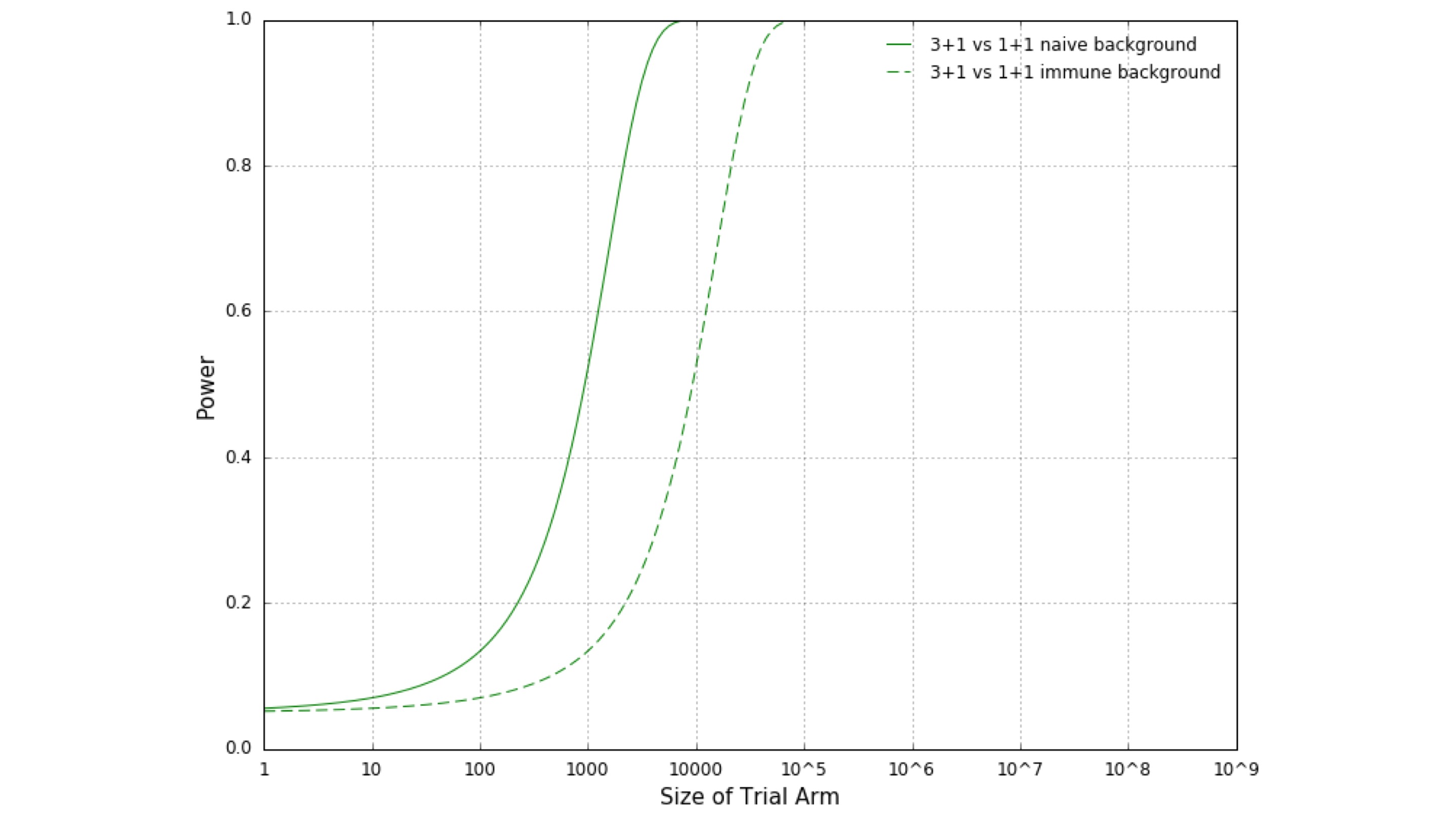

Supplement: Supplementary file 5 — Figure S4. Power vs. Trial Arm Size in a Simulated Trial of Goldblatt et al. PCV13. Simulated under assumptions of less divergence between the efficacies of different dose schedules. Sample sizes for a 3 + 1 vs 2 + 1 trial are not calculated because doing so would be incoherent; the arms are essentially identical, which implies an infinite theoretical sample size. (JPG 225 kb) [file 12879_2019_3833_MOESM5_ESM.jpg]
